# Firing frequency response to current and conductance periodic inputs in a I_h_/I_Nap_ biophysical neuron model

**DOI:** 10.1186/1471-2202-12-S1-P271

**Published:** 2011-07-18

**Authors:** Dongwook Kim, Horacio G Rotstein

**Affiliations:** 1 Department Mathematical Sciences, New Jersey Institute of Technology, Newark, NJ 07102, USA

## 

Various neurons exhibit membrane potential (or subthreshold) resonance [1], a peak in the subthreshold voltage amplitude response to oscillatory current inputs at a certain preferred (resonant) input frequency. Previous theoretical work using linear models has shown that subthreshold resonance can be communicated to the supra-threshold regime [2,3]; i.e., the firing frequency (or signal gain) response to oscillatory input currents peaks at the subthreshold resonant frequency. Whether this property is maintained under more general conditions and for different types of inputs is still an open question.

In this work, we investigate the firing frequency patterns generated in response to both current and conductance sinusoidal inputs in a biophysical (conductance-based) neuron model that includes two ionic currents: h- (I_h_) and persistent sodium (I_Nap_). This model is based on measurements for stellate cells (SCs) in layer II of the medial entorhinal cortex [4] and captures various phenomena observed in SCs such as membrane potential oscillations and membrane potential (subthreshold) resonance at theta frequencies (4 - 10 Hz)[2]. The I_h_/I_Nap_ model describes the cell's subthreshold dynamics and the onset of spikes [5]. Spikes were generated artificially as in integrate/resonate-and-fire models (see [5]).

We computed the firing rate and signal gain responses of the I_h_/I_Nap_ model to both current and conductance sinusoidal inputs for a wide range of frequencies and input amplitudes (A_in_). The resulting patterns for current and conductance input are qualitatively different. For current inputs, the firing frequency (and signal gain) patterns show up to three prominent peaks with heights that differ only slightly. The number of peaks increases with increasing values of A_in_. For small values of A_in_, the peak input frequency coincides with the subthreshold resonant frequency. The voltage traces (voltage vs. time) corresponding different peaks for the same value of A_in_ have roughly the same number of spikes (and inter-spike intervals of similar size), and differ in the number of subthreshold oscillations interspersed in between two consecutive spikes. For conductance (excitatory) inputs, the firing rate patterns show multiple peaks with different heights. As A_in_ increases, the highest peak moves to the right. For input frequencies above 60 Hz, the firing rate and gain of the highest peak significantly increases relative to other peaks for the same value of A_in_. This prominent peak reflects the fast time scale present in the model that has been shown to underlie hyper-excitable firing in SCs [6] observed in animal models of temporal lobe epilepsy.

We use dynamical systems tools to explain the mechanism that govern the generation of the firing frequency patterns described above. We show that the nonlinearities present in the model and the time scale separation between voltage and the h-current gating variables play an important role in determining these patterns.
